# Genome Editing in Cowpea *Vigna unguiculata* Using CRISPR-Cas9

**DOI:** 10.3390/ijms20102471

**Published:** 2019-05-19

**Authors:** Jie Ji, Chunyang Zhang, Zhongfeng Sun, Longlong Wang, Deqiang Duanmu, Qiuling Fan

**Affiliations:** 1 State Key Laboratory of Agricultural Microbiology, College of Life Science and Technology, Huazhong Agricultural University, Wuhan 430070, China; jieji920712@163.com (J.J.); zhangchunyang233@163.com (C.Z.); joson1987@163.com (Z.S.); llwang@webmail.hzau.edu.cn (L.W.); 2 College of Life Science and Technology, Huazhong Agricultural University, Wuhan 430070, China

**Keywords:** genome editing, CRISPR/Cas9, cowpea, symbiosis, nitrogen fixation

## Abstract

Cowpea (*Vigna unguiculata*) is widely cultivated across the world. Due to its symbiotic nitrogen fixation capability and many agronomically important traits, such as tolerance to low rainfall and low fertilization requirements, as well as its high nutrition and health benefits, cowpea is an important legume crop, especially in many semi-arid countries. However, research in *Vigna unguiculata* is dramatically hampered by the lack of mutant resources and efficient tools for gene inactivation in vivo. In this study, we used clustered regularly interspaced short palindromic repeats (CRISPR) and CRISPR-associated protein 9 (Cas9). We applied the CRISPR/Cas9-mediated genome editing technology to efficiently disrupt the representative symbiotic nitrogen fixation (SNF) gene in *Vigna unguiculata*. Our customized guide RNAs (gRNAs) targeting symbiosis receptor-like kinase (SYMRK) achieved ~67% mutagenic efficiency in hairy-root-transformed plants, and nodule formation was completely blocked in the mutants with both alleles disrupted. Various types of mutations were observed near the PAM region of the respective gRNA. These results demonstrate the applicability of the CRISPR/Cas9 system in *Vigna unguiculata*, and therefore should significantly stimulate functional genomics analyses of many important agronomical traits in this unique crop legume.

## 1. Introduction

**Figure 1:**
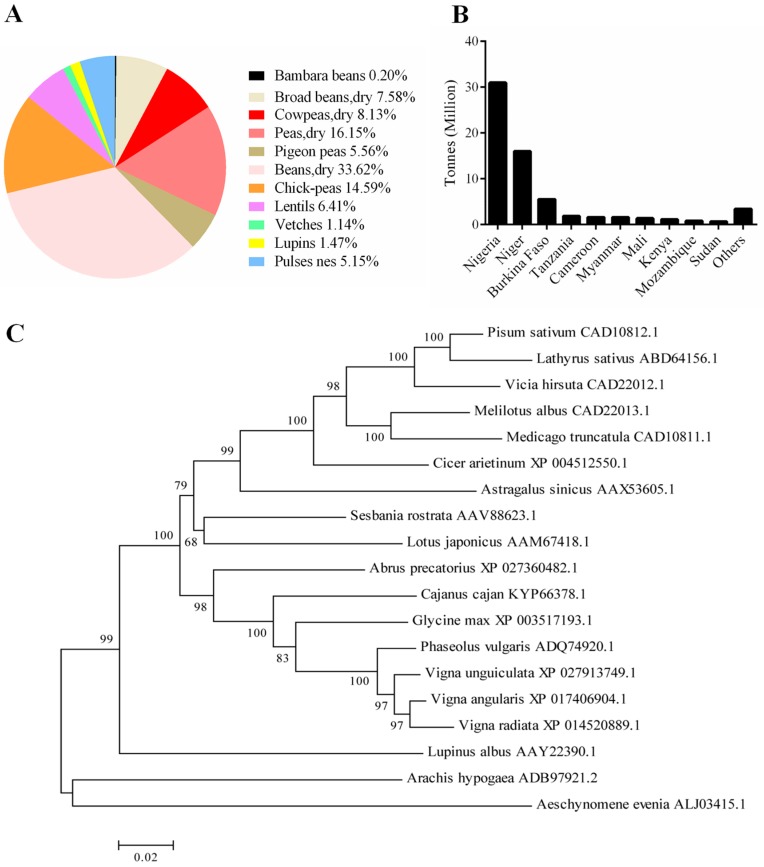
Global production of cowpea and legume SYMRK (symbiosis receptor-like kinase) phylogenetic analysis. (**A**) Percentages of worldwide dried seeds production of non-soybean legumes. (**B**) Top 10 countries with the largest cowpea production during 2008–2017 based on FAO (Food and Agriculture Organization of the United Nations). (**C**) Phylogenetic tree of SYMRK proteins in representative leguminous plants. The tree was constructed by MEGA 5 and with 1000 bootstrap replications. Protein accession numbers are from NCBI.

Cowpea (*Vigna unguiculata* L. Walp) belongs to the genus *Vigna* and originated in Africa. In this species, there are ten wild subspecies (ssp. *baoulensis*, *burundiensis*, *letozeyi*, *aduensis*, *pawekiae*, *dekindtiana, stenophylla*, *tenuis*, *alba*, and *pubescens*) and one domesticated subspecies (ssp. *unguiculata*) [1,2]. Only the ssp. *unguiculata* contains all the cultivar groups including *unguiculata, biflora, sesquipedalis*, *textilis*, and *melanophthalmus* [3]. In the last decade (2008–2017), among the 11 primary legumes harvested as dry seeds, cowpea was ranked the 4th highest production of dry grain across the world [4] (Figure 1A). More importantly, cowpea is a widely cultivated food grain, especially in semi-arid tropical regions. In many African countries, cowpea is a significant source of cheap protein for human health due to its excellent capability of drought tolerance and growth in low-nutrient soils. Nigeria and Niger are the two largest cowpea producing countries, accounting for ~47.6% and ~24.6% of the global production, respectively (Figure 1B). Additionally, cowpea leaves can also be used as livestock feed in many developing countries. 

In barren soil, cowpea is able to establish symbiotic associations with nitrogen-fixing bacteria and phosphorus-absorbing arbuscular mycorrhizal fungi. Cowpea could form nodules upon inoculation with *Sinorhizobium*, *Rhizobium*, and *Bradyrhizobium* [5,6,7]. *Rhizobium* sp. strain NGR234 has a broad host range and was shown to be able to induce nodules on more than 232 legume species including *Vigna unguiculata* [5,6,8]. However, our understanding of the molecular mechanism of symbiotic nitrogen fixation (SNF) during cowpea–rhizobia interactions is still in its infancy. Indeed, research on cowpea has lagged far behind other legumes with sequenced genomes, such as *Glycine max*, *Medicago truncatula,* and *Lotus japonicus* [9]. However, more information on the genetic sequencing of cowpea is now available due to the increase in interest and research on this legume. The HarvEST:Cowpea (https://harvest.ucr.edu/) contains a consensus genetic map with over 180,000 expressed sequence tags (ESTs), over 4000 bacterial artificial chromosome (BAC) sequence assembles, and ~29,000 unigene consensus sequences [10]. By combining genetic resources from 37 cowpea accessions, a consensus genetic map with 37,372 single nucleotide polymorphisms (SNPs) was recently constructed [11]. The sequencing and assembly of the *Vigna unguiculata* draft genome predicted a nuclear genome containing ~42,287 protein-coding loci (*Vigna unguiculata* v1.0, NSF, UCR, USAID, DOE-JGI, http://phytozome.jgi.doe.gov). However, the lack of appropriate mutant resources in *Vigna unguiculata* severely restricts the development of this climate-resilient legume as a model species for basic research and plant breeding studies.

Sequence-specific nucleases (SSNs)-based genome editing technologies have rapidly evolved in the last decade and revolutionized life science research in all fields. Compared with the earlier developed zinc finger nucleases (ZFNs) and transcription activator-like effector nucleases (TALENs), the clustered regularly interspaced short palindromic repeats (CRISPR) associated systems utilized a guide RNA (usually 20 nt) for specific recognition of the target gene [12,13,14,15]. Because of the efficacy, feasibility, and resource availability, CRISPR-associated protein 9 (Cas9)-mediated genome editing has been adopted as a convenient and powerful tool for gene manipulations in dozens of model plants and crop species [16,17]. Among legumes, nuclear genes of *L. japonicus* [18], *G. max* [19], and *M. truncatula* [20] were all successfully edited by CRISPR-Cas9.

In this study, we successfully applied the CRISPR-Cas9 system to disrupt the symbiosis receptor-like kinase (*SYMRK*) gene in *Vigna unguiculata*. *SYMRK* is indispensable for both nodule and arbuscular mycorrhizal symbiosis, and is conserved in legumes including *L. japonicus*, *M. sativa*, *M. truncatula*, *Pisum sativum,* and *G. max* [21,22,23]. We identified a homologous *SYMRK* gene in cowpea and named this gene *VuSYMRK*. Three guide RNAs targeting exon 2 of *VuSYMRK* were designed by CRISPR-P2.0 and knockout mutants were created by *Agrobacterium rhizogenes* K599-mediated hairy root transformation. Mutant plants failed to develop nodules upon inoculation with *Sinorhizobium* sp. strain NGR234, corroborating the essential function of *VuSYMRK* in the establishment of nodule symbiosis in *Vigna unguiculata*.

## 2. Results

To identify the SYMRK homologous protein in cowpea, we used common bean (*Phaseolus vulgaris*) SYMRK protein sequence (NCBI accession: ADQ74920.1) as the query and identified a single copy homologous protein by BLASTp. A phylogenetic tree of 19 SYMRK proteins from 19 legume species was constructed using the MEGA 5 software (www.megasoftware.net) (Figure 1C). Cowpea SYMRK protein (VuSYMRK) showed the highest homology with *Vigna radiata* and *Vigna angularis* SYMRKs. These three Vigna SYMRK proteins were clustered together with common bean (*P. vulgaris*) and soybean (*G. max*), and separated from the other cluster comprising *Arachis hypogaea* and *Aeschynomene evenia*. The evolutionary relatedness of SYMRK proteins is consistent with the phylogeny and estimated divergence times of the phaseoloid legumes, including soybean, common bean, and cowpea [5], whereas *Arachis hypogaea* and *Aeschynomene evenia* both belong to Dalbergieae of the Faboideae subfamily.

**Figure 2:**
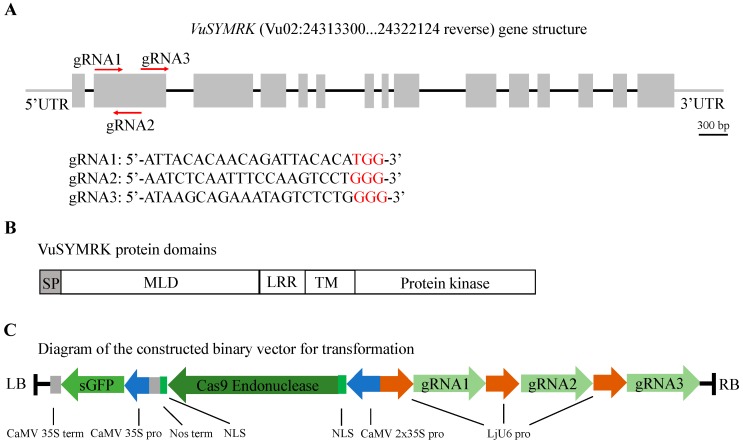
Schematic diagram of the symbiosis receptor-like kinase (*VuSYMRK*) gene structure, protein domains and the CRISPR-associated protein 9 (Cas9)/guide RNA (gRNA) binary vector. (**A**) Diagram of the *VuSYMRK* gene structure. The *VuSYMRK* gene is located on the reverse strand of chromosome 2 from 24313300 to 24322124. Grey line, untranslated region (UTR); black line, introns; grey box, exons; red arrows, gRNA target sites. The PAM sequences are in red font. (**B**) Diagram of predicted protein domains. SP, signal peptide; MLD, malectin-like domain; LRR, leucine-rich repeat; TM, transmembrane domain. (**C**) Illustration of the constructed binary vector for the CRISPR-Cas9 system. RB and LB, right and left borders; CaMV 35S pro, Cauliflower mosaic virus (CaMV) 35S promoter; CaMV 2X35S pro, CaMV 2X35S promoter; CaMV 35S term, CaMV 35S terminator; Nos term, nopaline synthase gene (Nos) terminator; NLS, nuclear localization sequence; LjU6 pro, *L. japonicus* U6 promoter.

Similar to other legume SYMRK proteins, VuSYMRK contains a predicted malectin-like domain (MLD), three leucine-rich repeats (LRRs), a transmembrane domain (TM), and the intracellular kinase domain (Figure 2B). To validate the *VuSYMRK* gene sequence, we designed gene specific primers, VuSYMRK-exon 1-F and VuSYMRK-exon 3-R (sequences of the primers can be found in Table S1) to amplify the region (~1972 bp) between exon 1 and exon 3 of the *VuSYMRK* gene. Sequencing results indicate that the sequence of this region is the same as the sequence from Phytozome (Vigun02g088500). Three gene-specific guide RNAs were then designed by CRISPR-P2.0 (http://crispr.hzau.edu.cn/CRISPR2) to target exon 2 (corresponding to partial MLD domain) of *VuSYMRK*. To enhance the gene disruption efficiency, these three gRNAs were constructed into one binary vector, under the control of *L. japonicus* U6 small nuclear RNA promoter (LjU6 pro), with a co-expression of the Cas9 endonuclease and a GFP fluorescent marker [18] (Figure 2C).

**Figure 3:**
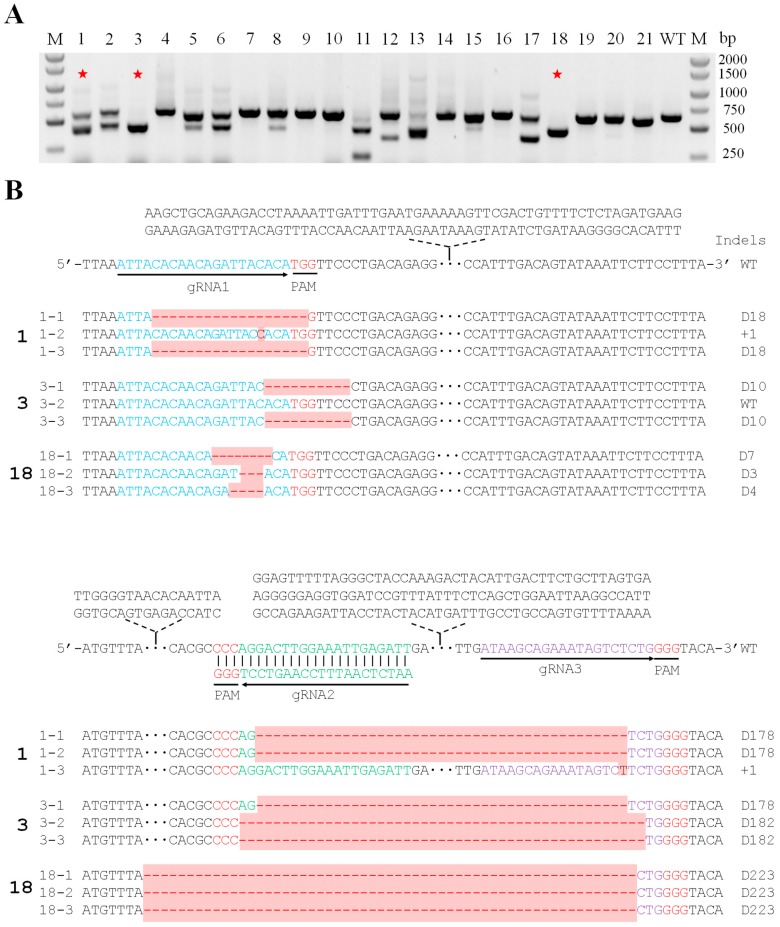
CRISPR/Cas9-induced mutations on the *VuSYMRK* gene. (**A**) PCR detection of CRISPR/Cas9-induced mutations of the *VuSYMRK*. Lanes 1–21, sequence amplified from genomic DNA isolated from hairy root transgenic cowpea plants by primers (SYMRK-gDNA-F/R). M, DNA marker; WT, wild-type plant; red star, sequenced transgenic lines. (**B**) Mutations between gRNA target sites on *VuSYMRK* gene. blue font, gRNA1; green font, gRNA2; purple font, gRNA3; red font, PAM sequence; D3/4/7/10/18/178/182/223, nucleotide deletions; +1, 1 bp insertion; DNA insertions or deletions are highlighted in red.

*A. rhizogenes* K599 containing *VuSYMRK*-gRNA1/2/3 or empty vector was used for the hairy root transformation of cowpea. Positive transgenic roots were identified by fluorescence observation under a light microscope. Genomic DNA of 21 GFP-positive transgenic hairy roots was extracted for PCR assay by primers spanning the three gRNA target sites (SYMRK-gDNA-F/R) to detect CRISPR/Cas9-induced mutations on *VuSYMRK* (Figure 3A). The wild-type plant gave one PCR product around 640 bp. Among the 21 different lines, the band size was either close to or different from the wild type, indicating the presence of minor or large additions or deletions. Some lines produced only one band, and the others contained bands of different sizes. Among these 21 lines, we chose three transgenic lines (lines 1, 3, 18) to validate *VuSYMRK* nucleotide sequence at the gRNA sites. PCR fragments of these three lines were subcloned into a vector (pEASY T5-zero) and more than 5 individual *E. coli* colonies were picked up for plasmid extraction and sequencing. As expected, sequencing results showed that the *VuSYMRK* in each line exhibited various mutations. Both small scale nucleotide insertion/deletion (between gRNA1 and gRNA2) and insertion/large fragment deletion (between gRNA2 and gRNA3) were observed (Figure 3B). No wild-type allele was present in these lines, and each line contained at least three types of allelic mutations, most likely due to the chimeric status of the regenerated hairy roots. Overall, these results demonstrated that the CRISPR/Cas9 system was effective in causing gene mutations in cowpea.

**Figure 4:**
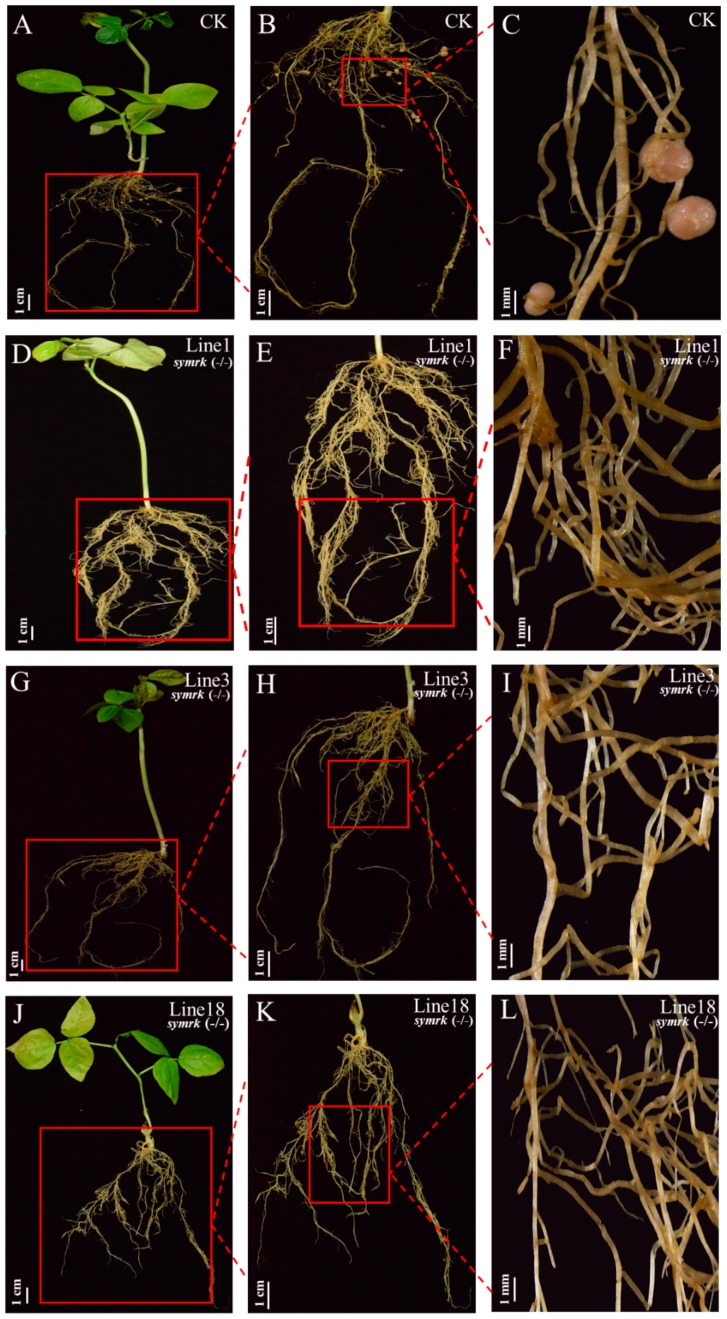
Symbiotic nodulation phenotype of cowpea *SYMRK* mutants. The transgenic hairy roots were inoculated with *Sinorhizobium* sp. NGR234. Pictures were taken after three weeks post inoculation (3 wpi). (**A**–**C**) Nodulation phenotype of hairy roots transformed with the empty vector (CK): (**A**) whole plant; (**B**) hairy roots; (**C**) enlarged hairy roots with nodules. (**D**–**L**) Nodulation phenotype of *SYMRK* mutant line 1 (**D**–**F**), line 3 (**G**–**I**) and line 18 (**J**–**L**).

We then compared the symbiotic nodulation phenotype of these mutated plants by inoculating the transformed hairy roots with *Sinorhizobium* sp. NGR234. After three weeks post inoculation (3 wpi), the hairy roots that were transformed with empty vector developed typical mature nodules with pink color (Figure 4A–C), whereas line 1, line 3, and line 18 had no detectable nodule primordia or nodules (Figure 4D–L). Since both alleles of *VuSYMRK* were disrupted in lines 1, 3, and 18, the observed symbiotic deficiency phenotype indicates that *VuSYMRK* is indispensable for the nodule organogenesis in cowpea.

**Figure 5:**
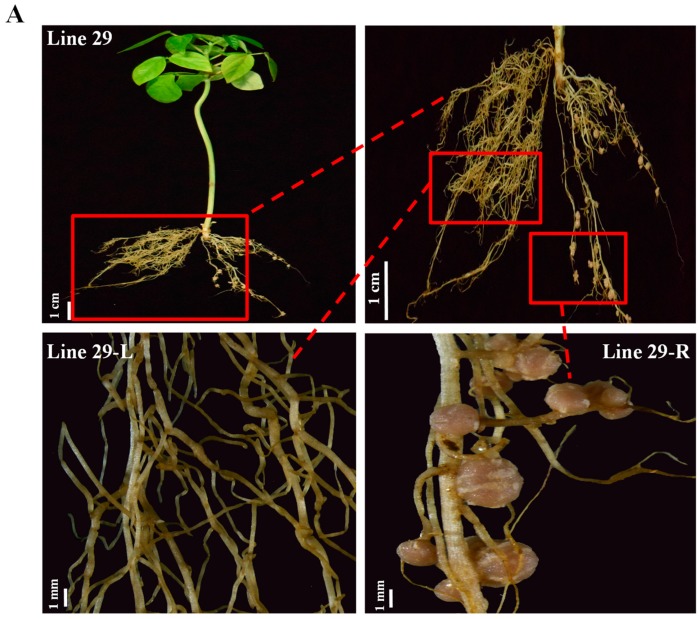

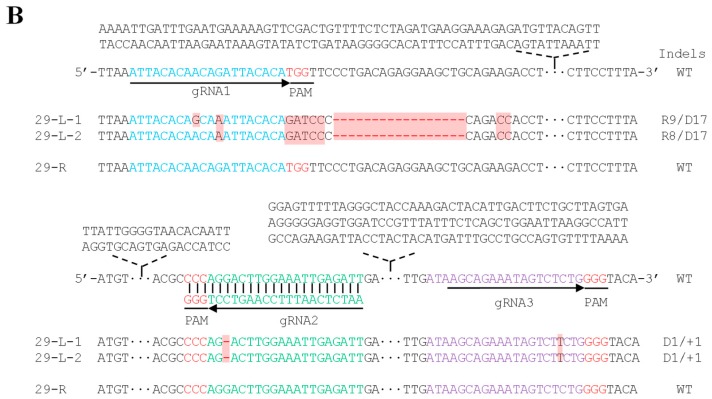
Nodulation phenotype and sequencing of *SYMRK* in a split-root system. The transgenic hairy roots were inoculated with *Sinorhizobium* sp. NGR234. Pictures were taken after 3 weeks post inoculation (3 wpi). (**A**) Nodulation phenotype of hairy roots. Line 29-L, left part root of line 29; Line 29-R, right part root of line 29. (**B**) Mutations around gRNA target sites of *VuSYMRK* gene. Blue font, gRNA1; green font, gRNA2; purple font, gRNA3; red font, PAM sequence; WT, wild-type sequence; D1/17, 1 bp or 17 bp nucleotide deletions; R8/9, 8 bp or 9 bp nucleotide replacements; +1, 1 bp insertion; DNA replacements, insertions and deletions are highlighted in red.

*A. rhizogenes* K599 incited hairy roots near the cotyledon region and a number of hairy roots could be induced from one transformed plant. Most of the transformed lines (Line 1~21) produced several roots, and we kept only one main root and removed the other hairy roots for nodulation assay. To ascertain if roots evolved from the same transgenic plant could harbor different genetic background and exhibit distinct phenotypes, we kept two hairy roots and assayed the nodulation phenotype at three weeks post inoculation with *Sinorhizobium* sp. NGR234. Interestingly in Line 29, one hairy root (Line 29-R) produced well-developed nodules, whereas no nodules were observed on the other hairy root (Line 29-L) (Figure 5A). Sequencing analyses demonstrated that the root 29-R only contained a wild-type sequence, whereas 29-L had two types of mutated *SYMRK* alleles, all leading to null mutation of the gene (Figure 5B). These results indicate that hairy root transformation could result in different genetic backgrounds in the regenerated hairy roots and therefore could enable root-specific phenotypic comparisons under the same shoot genotype. 

We last investigated whether or not the potential off-target sites of these three gRNAs were also affected in these transgenic hairy roots. The top three potential off-target sites were selected for each gRNA. For each line, we analyzed the nine total potential off-target sites. Corresponding PCR primers (Table S1) were designed to amplify the flanking regions of these nine sites. Sequencing results confirmed that there were no mutations in lines 1, 3, 18, 29 for all nine potential off-target sites (Figure S1). Therefore, these putative off-target sites were not edited in vivo.

## 3. Discussion

SYMRK is essential for nodule formation in *Lotus japonicus* and *Glycine max*. SYMRK contains four domains including MLD, LRR, transmembrane domain, and protein kinase domain. MLD negatively interferes with the complex formation between SYMRK and Nod factor receptor 5 (NFR5). The LRR domain promotes interaction with the NFR5 ectodomain and also stimulates SYMRK protein degradation, whereas the kinase domain is responsible for symbiotic signal transduction [22,23]. In this study, cowpea *SYMRK* gene was successfully edited by the CRISPR-Cas9 system, and nodule formation was completely inhibited in the mutants when both two alleles were disrupted. In order to achieve the higher gene editing efficiency, three gRNAs targeting both DNA strands at different sites of *VuSYMRK* exon 2 were used and we observed various indels in *SYMRK* gene, including large fragment deletion between target sites of gRNA2 and gRNA3. 

The overall high efficiency of gene editing observed in this study suggests that simultaneous application of multiple gRNAs is a reasonable approach to achieve satisfactory gene editing efficiency and could facilitate PCR detection of large fragment deletions. 

Besides Cas9, other CRISPR systems have been recently developed and resulted in higher efficiency and lower off-target effects in many animal and plant species [24,25,26]. One example is Cpf1, another type of CRISPR nuclease. Compared to Cas9, Cpf1 has the ability to target specific sites of genome and it breaks double-stranded DNA near the T-rich PAM, forming staggered ends to help DNA sequence insertion. The CRISPR–Cpf1 system was also shown to have much lower off-target effects than Cas9 [27]. Due to the advantages of CRISPR–Cpf1, it could be an alternative system to edit specific cowpea genes to facilitate functional genomics research in cowpea in the future. 

Construction of stable transgenic mutant plants with specific gene modifications is the next goal of application of genome editing technology in cowpea. In this study, we used the hairy root transformation approach and observed the presence of different types of *VuSYMRK* mutant alleles in the regenerated hairy roots. *A. rhizogenes* is the causal agent of hairy root disease. It contains Ri (root-inducing) plasmid and has the ability to transfer and integrate a region of Ri into the plant nuclear genome, acting similarly as *A. tumefaciens* containing Ti plasmid [28,29]. *A. rhizogenes*-mediated hairy root transformation has been widely used in recalcitrant plant species for heterologous gene expression and study of gene function associated with roots [30]. *A. rhizogenes* strain K599 has been used to incite hairy roots from cotyledons of soybean and common bean [31,32,33]. In this study, we established the cowpea hairy root transformation procedure by *A. rhizogenes* strain K599. Stable transformation in cowpea is currently still technically challenging, i.e., only ~1% transformation efficiency was achieved using cotyledonary nodes derived explants and a stringent selection regime [34]. The widely applied transformation technologies in plants include pollen tube pathway [35], *Agrobacterium*-mediated transformation [36] and particle bombardment [37]. Establishment of an efficient stable transformation system in cowpea has therefore become an urgent need at this stage.

## 4. Materials and Methods 

### 4.1. Plant Materials and Growth Conditions

Cowpea (*Vigna unguiculata*) were sowed in autoclaved vermiculite and perlite (2:1) and maintained in a plant growth room of 16 h light/8 h dark at 26 °C under cool white fluorescent light with an intensity of 60~100 μmol photons m^−2^·s^−1^. 

### 4.2. Vector Construction

Using *Phaseolus vulgaris* symbiosis receptor-like kinase (SYMRK) protein sequence (NCBI GenBank: ADQ74920.1) as the query, a homologous protein (Phytozome reference Vigun02g088500) could be identified in cowpea genome (*Vigna unguiculata* v1.0, NSF, UCR, USAID, DOE-JGI, http://phytozome.jgi.doe.gov/). This gene was named as *VuSYMRK*. Three guide RNAs (gRNA1, gRNA2, gRNA3) were selected by the web tool CRISPR-P2.0 (http://crispr.hzau.edu.cn/cgi-bin/CRISPR2) [38]. Three primer pairs were designed as VuSYMRK-gRNA1-BbsI-F/R, VuSYMRK-gRNA2-BbsI-F/R, and VuSYMRK-gRNA3-BbsI-F/R (sequences of the primers can be found in Table S1). Three gRNAs were produced by denaturing forward and reverse primer pairs at 95 °C for 4 min, followed by a temperature decrease of 0.1 °C/s to 35 °C and then incubation at 35 °C for 30 s. Then double stranded gRNA1, gRNA2, and gRNA3 were individually ligated into the vector pB-LjU6 [18] predigested by BbsI, resulting in pB-LjU6-VuSYMRK-gRNA1, pB-LjU6-VuSYMRK-gRNA2, and pB-LjU6-VuSYMRK-gRNA3, respectively. Plasmids containing gRNA1 and gRNA2 were digested by KpnI and XbaI, releasing LjU6-VuSYMRK-gRNA1 and LjU6-VuSYMRK-gRNA2. Fragment LjU6-VuSYMRK-gRNA2 was ligated with pB-LjU6-VuSYMRK-gRNA3 cut by KpnI and SpeI, creating pB-LjU6-VuSYMRK-gRNA2+3. LjU6-VuSYMRK-gRNA1 was subcloned into pB-LjU6-VuSYMRK-gRNA2+3 using KpnI and SpeI sites, resulting in pB-LjU6-VuSYMRK-gRNA1+2+3. This plasmid was then cut by KpnI and XbaI, releasing LjU6-VuSYMRK-gRNA1+2+3 fragment, which was subcloned into pCAMBIA1300-35S-sGFP-2X35S-Cas9 [18], resulting in the final construct pCAMBIA1300-35S-sGFP-2X35S-Cas9-LjU6-VuSYMRK-gRNA1+2+3. All restriction enzymes were purchased from Fermentas.

### 4.3. Cowpea Hairy Root Transformation

Appropriate plasmids were electroporated into competent *A. rhizogenes* K599 cells. The positive colonies were verified by colony PCR with primers SYMRK-gDNA-F/R (sequences of the primers can be found in Table S1). Transformed K599 cells were maintained on LB plate with kanamycin (50 mg/L) and streptomycin (50 mg/L) and incubated at 28 °C overnight. Then the *Agrobacterium* lawn was collected and suspended in 1 mL 30% glycerol. The suspension was spread onto a new YEP (Tryptone 10 g/L, Yeast Extract 10 g/L, NaCl 5 g/L, Agar 2%) plate with kanamycin (50 mg/L), streptomycin (50 mg/L), and acetosyringone (40 mg/L), and the plate was incubated at 28 °C overnight. Bacterial lawn was harvested and used for plant transformation. Cowpea seedlings (3- or 4-day-old) were chosen to be inoculated with *A. rhizogenes* K599. At this stage, cowpea primary leaves were enclosed within or just reached out of the cotyledons. A syringe needle was used to make holes just below 1 cm of cotyledonary nodes. *A. rhizogenes* K599 transformed with pCAMBIA1300-35S-sGFP-2X35S-Cas9-LjU6-VuSYMRK-gRNA1+2+3 or the empty vector were transferred to the wounds by syringe needles. After inoculation with *Agrobacterium rhizogenes* K599, plants below the cotyledonary nodes were covered by wet vermiculite and perlite and were kept in a tray with a transparent lid to maintain the humidity between 70% and ~90%. The bottom of the tray was filled with water and the humidity was checked daily until the hairy roots evolved. When the hairy roots reached ~2 cm length, the original roots were removed and one hairy root was kept. Seedlings with transgenic hairy roots were transferred to a new pot with vermiculite and perlite (2:1), covered with a transparent lid for 3 days. The lid was removed until the seedlings were recovered.

### 4.4. Rhizobia Inoculation and Phenotypic Analysis 

The *Sinorhizobium* sp. NGR234 cells from a frozen stock were streaked on a TY plate (TY: yeast extract 3 g/L, tryptone 5 g/L, CaCl_2_·2H2O 0.91 g/L, Agar 1.8%) and maintained at 28 °C for 3–5 days. Rhizobia cells were transferred to TY medium and grew at 28 °C and 200 rpm until OD_600_ reached 0.4–0.6. Cells were collected by centrifugation at 4000 rpm for 15 min and then resuspended in 1 L Broughton and Dilworth (B&D) medium [39] (supplemented with 0.5 mM KNO_3_) to OD_600_ of 0.02–0.03. The suspension cells were used to inoculate transgenic plants. After inoculation, plans were maintained in autoclaved vermiculite and perlite (2:1) and irrigated with one-half-strength Broughton and Dilworth nutrient solution without any source of nitrate. After three weeks of inoculation, nodules on hairy roots were compared under a light microscope. 

### 4.5. DNA Isolation, PCR, Plasmid Extraction and Sequencing

Genomic DNA was extracted by using NuClean Plant Genomic DNA Kit (CWBio, Beijing, China). PCR reactions were performed by specific primers using standard procedures. The PCR products were sequenced by specific primers. If the sequencing results contained mutations, the PCR band was extracted from the agarose gel, then purified and cloned into pEASY T5-ZERO vector (TransGen Biotech, Beijing, China). Five individual *E. coli* colonies were selected for plasmid preparation (TIANprep Mini Plasmid Kit, TIANGEN, Beijing, China) and sequencing by Sanger method.

## 5. Conclusions

In this study, we developed an efficient CRISPR-Cas9 system for symbiosis receptor-like kinase gene inactivation in cowpea by means of hairy root transformation mediated by *Agrobacterium rhizogenes* K599. CRISPR/Cas9-mediated genome editing technology effectively disrupted this essential SNF gene in cowpea. To our knowledge, this is the first report of successful application of genome editing technology in cowpea. We expect that this technology should be able to dramatically stimulate functional genomics analyses of many agronomically important traits in this special type of legume. 

## Supplementary Materials

The following are available online at https://www.mdpi.com/1422-0067/20/10/2471/s1.

## Abbreviations

BAC	bacterial artificial chromosome
Cas9	CRISPR-associated protein 9
Cpf1	CRISPR-associated protein, subtype Prevotella and Francisella
CRISPR	clustered regularly interspaced short palindromic repeats
ESTs	expressed sequence tags
gRNA	guide RNA
LRR	leucine-rich repeats
MEGA	molecular evolutionary and genetics analysis
PAM	protospacer adjacent motif
Ri	root-inducing
SNF	symbiotic nitrogen fixation
SNPs	single nucleotide polymorphisms
SSNs	sequence-specific nucleases
SYMRK	symbiosis receptor-like kinase
TALENs	transcription activator-like effector nucleases
Ti	tumor-inducing
YEP	yeast extract peptone
ZFNs	zinc finger nucleases
