# Supplementary material for: Genome Editing in Cowpea Vigna unguiculata Using CRISPR-Cas9
Source: Int J Mol Sci. 2019 May 19;20(10):2471. doi: 10.3390/ijms20102471 (PMC6566367; doi:10.3390/ijms20102471)
Supplement: Supplementary file 1 [file ijms-20-02471-s001.pdf]

A

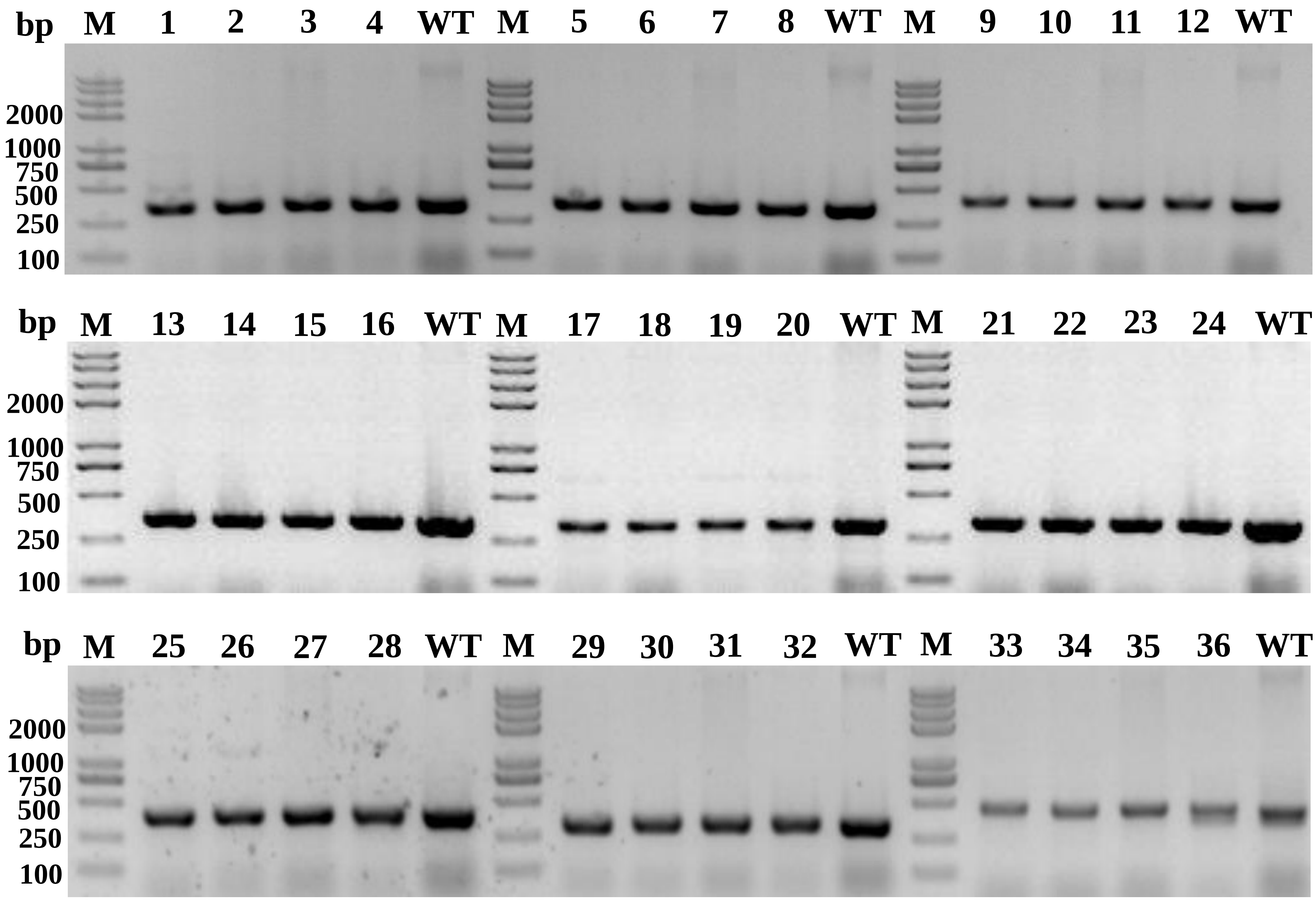

B

gRNA1 off-target sites

Vigun05g271100

gRNA1 off-target site 1

WT 5'—CTGGCTTACTGCTACATTAAACTAAATATTACACAAGGTAGAGCGCTTCCTGA—3'

Line1 CTGGCTTACTGCTACATTAAACTAAATATTACACAAGGTAGAGCGCTTCCTGA

Line3 CTGGCTTACTGCTACATTAAACTAAATATTACACAAGGTAGAGCGCTTCCTGA

Line18 CTGGCTTACTGCTACATTAAACTAAATATTACACAAGGTAGAGCGCTTCCTGA

Line29 CTGGCTTACTGCTACATTAAACTAAATATTACACAAGGTAGAGCGCTTCCTGA

Vigun11g100700

gRNA1 off-target site 2

WT 5'—AAAGGAAAAGGGAAAATATCAAAACACATTACACATGGCTTCCACTTGATGAA—3'

Line1 AAAGGAAAAGGGAAAATATCAAAACACATTACACATGGCTTCCACTTGATGAA

Line3 AAAGGAAAAGGGAAAATATCAAAACACATTACACATGGCTTCCACTTGATGAA

Line18 AAAGGAAAAGGGAAAATATCAAAACACATTACACATGGCTTCCACTTGATGAA

Line29 AAAGGAAAAGGGAAAATATCAAAACACATTACACATGGCTTCCACTTGATGAA

Vigun07g201000

gRNA1 off-target site 3

WT 5'—CATGCAAATAAATACATTCATGAACAGATTACACAAGGTAAATAAACAGTACC—3'

Line1 CATGCAAATAAATACATTCATGAACAGATTACACAAGGTAAATAAACAGTACC

Line3 CATGCAAATAAATACATTCATGAACAGATTACACAAGGTAAATAAACAGTACC

Line18 CATGCAAATAAATACATTCATGAACAGATTACACAAGGTAAATAAACAGTACC

Line29 CATGCAAATAAATACATTCATGAACAGATTACACAAGGTAAATAAACAGTACC

gRNA2 off-target sites

Vigun03g327900

gRNA2 off-target site 1 →

**WT**	5'—CAGACCTCCGCATCGAATGGCATTCTCCAAGTCCTTGGACTCCGCAACGAAAA—3'
**Line1**	CAGACCTCCGCATCGAATGGCATTCTCCAAGTCCTTGGACTCCGCAACGAAAA
**Line3**	CAGACCTCCGCATCGAATGGCATTCTCCAAGTCCTTGGACTCCGCAACGAAAA
**Line18**	CAGACCTCCGCATCGAATGGCATTCTCCAAGTCCTTGGACTCCGCAACGAAAA
**Line29**	CAGACCTCCGCATCGAATGGCATTCTCCAAGTCCTTGGACTCCGCAACGAAAA

Vigun08g093300

gRNA2 off-target site 2 →

**WT**	5'—ACGAAAGTGCTCATCAATTTTAATTTCCAAGTTCTGAGGAACAAAAGGTAATT—3'
**Line1**	ACGAAAGTGCTCATCAATTTTAATTTCCAAGTTCTGAGGAACAAAAGGTAATT
**Line3**	ACGAAAGTGCTCATCAATTTTAATTTCCAAGTTCTGAGGAACAAAAGGTAATT
**Line18**	ACGAAAGTGCTCATCAATTTTAATTTCCAAGTTCTGAGGAACAAAAGGTAATT
**Line29**	ACGAAAGTGCTCATCAATTTTAATTTCCAAGTTCTGAGGAACAAAAGGTAATT

Vigun09g252400

gRNA2 off-target site 3 →

**WT**	5'—AATTTGCAGACGGTTCATCTCGGTTTCCATGTCCTGGGCTCTTGAAACACGTG—3'
**Line1**	AATTTGCAGACGGTTCATCTCGGTTTCCATGTCCTGGGCTCTTGAAACACGTG
**Line3**	AATTTGCAGACGGTTCATCTCGGTTTCCATGTCCTGGGCTCTTGAAACACGTG
**Line18**	AATTTGCAGACGGTTCATCTCGGTTTCCATGTCCTGGGCTCTTGAAACACGTG
**Line29**	AATTTGCAGACGGTTCATCTCGGTTTCCATGTCCTGGGCTCTTGAAACACGTG

gRNA3 off-target sites

Vigun03g042200

gRNA3 off-target site 1 →

**WT**	5'—AGGAAAATGATGTGCAAAAGCAGAAGCAGTCTCTGAAGAACAAGGCGTGGGAA—3'
**Line1**	AGGAAAATGATGTGCAAAAGCAGAAGCAGTCTCTGAAGAACAAGGCGTGGGAA
**Line3**	AGGAAAATGATGTGCAAAAGCAGAAGCAGTCTCTGAAGAACAAGGCGTGGGAA
**Line18**	AGGAAAATGATGTGCAAAAGCAGAAGCAGTCTCTGAAGAACAAGGCGTGGGAA
**Line29**	AGGAAAATGATGTGCAAAAGCAGAAGCAGTCTCTGAAGAACAAGGCGTGGGAA

Vigun03g308200

gRNA3 off-target site 2 →

**WT**	5'—TACTGTGACATGGAAATAAGTAGTAATAATCACTGGGGGTGATCTATATTTTT—3'
**Line1**	TACTGTGACATGGAAATAAGTAGTAATAATCACTGGGGGTGATCTATATTTTT
**Line3**	TACTGTGACATGGAAATAAGTAGTAATAATCACTGGGGGTGATCTATATTTTT
**Line18**	TACTGTGACATGGAAATAAGTAGTAATAATCACTGGGGGTGATCTATATTTTT
**Line29**	TACTGTGACATGGAAATAAGTAGTAATAATCACTGGGGGTGATCTATATTTTT

Vigun02g034200

gRNA3 off-target site 3 →

**WT**	5'—ACCATAAAAAATTACATAATTAGAAATATTTTCTGGAGCATTATTGATTAAGA—3'
**Line1**	ACCATAAAAAATTACATAATTAGAAATATTTTCTGGAGCATTATTGATTAAGA
**Line3**	ACCATAAAAAATTACATAATTAGAAATATTTTCTGGAGCATTATTGATTAAGA
**Line18**	ACCATAAAAAATTACATAATTAGAAATATTTTCTGGAGCATTATTGATTAAGA
**Line29**	ACCATAAAAAATTACATAATTAGAAATATTTTCTGGAGCATTATTGATTAAGA

Figure S1. Analysis of potential off-target sites of VuSYMRK gRNAs. **(A)** PCR detection of potential off-target sites of the designed three gRNAs. Lanes 1-4, gRNA1 off-target site 1 (Vigun05g271100) of lines 1, 3, 18, 29; Lanes 5-8, gRNA1 off-target site 2 (Vigun11g100700) of corresponding lines; Lanes 9-12, gRNA1 off-target site 3 (Vigun07g201000) of corresponding lines; Lanes 13-16, gRNA2 off-target site1 (Vigun03g327900); Lanes 17-20, gRNA2 off-target site 2 (Vigun08g093300); Lanes 21-24, gRNA2 off-target site 3 (Vigun09g252400); Lanes 25-28, gRNA3 off-target site 1 (Vigun03g042200); Lanes 29-32, gRNA3 off-target site 2 (Vigun03g308200); Lanes 33-36, gRNA3 off-target site 3 (Vigun02g034200). **(B)** Sequencing results of potential off-target sites in transgenic roots compared to the wild-type root (WT).

Table S1. Primers used in this study

Primer Name	Sequence (5' to 3')
VuSYMRK-exon1-F	ATGATGGAGTTACCAGAAATTTGGG
VuSYMRK-exon3-R	CATCTTTTTGGTTGGTCTCTTC
gRNA1	ATTACACAACAGATTACACATGG
gRNA2	AATCTCAATTTCCAAGTCCTGGGG
gRNA3	ATAAGCAGAAATAGTCTCTGGGG
VuSYMRK-gRNA1-BbsI-F	GTTCGATTACACAACAGATTACACA
VuSYMRK-gRNA1-BbsI-R	AAACTGTGTAATCTGTTGTGTAATC
VuSYMRK-gRNA2-BbsI-F	GTTCGATCTCAATTTCCAAGTCCTG
VuSYMRK-gRNA2-BbsI-R	AAACCAGGACTTGGAAATTGAGATC
VuSYMRK-gRNA3-BbsI-F	GTTCGATAAGCAGAAATAGTCTCTG
VuSYMRK-gRNA3-BbsI-R	AAACCAGAGACTATTTCTGCTTATC
SYMRK-gDNA-F	GGGAACATCCCTTTTAGTAC
SYMRK-gDNA-R	CCTAAGCCTTGTAGATTTC
symrk-gRNA1-off-target-1-F	GAGCGATCAAACAGCTGC
symrk-gRNA1-off-target-1-R	ACCTATTCCGGCAAAACC
symrk-gRNA1-off-target-2-F	GCAACTTGGATGTTCTTTC
symrk-gRNA1-off-target-2-R	CAACATCATGGCCTAAGTG
symrk-gRNA1-off-target-3-F	GATTATTTCCCACTGTGC
symrk-gRNA1-off-target-3-R	TGTGAACATTCTGCAGTG
symrk-gRNA2-off-target-1-F	CACCACCGAGACCAATTC
symrk-gRNA2-off-target-1-R	CTGTTTTGGGACCAATTCC
symrk-gRNA2-off-target-2-F	CGTCTCAGTCAAACTTCTC
symrk-gRNA2-off-target-2-R	GACTCAAAATTTCTCCACTC
symrk-gRNA2-off-target-3-F	CCAAGTCTCAGTTGCTAGAG
symrk-gRNA2-off-target-3-R	GGGTTGCAACGCATGATTTC
symrk-gRNA3-off-target-1-F	CAATGCTCAACAGCAGCAAC
symrk-gRNA3-off-target-1-R	CGGAAGATATTTCCTTCCC
symrk-gRNA3-off-target-2-F	CTTCCAGTATGTTGCGGG
symrk-gRNA3-off-target-2-R	GCCATTACTACCGCTAGC
symrk-gRNA3-off-target-3-F	GTAGTGCAGTGCAGTTTGG
symrk-gRNA3-off-target-3-R	GAACTCGTCCATGTCTGAG
